# Enhancing the Detection of *Brucella*-Specific CD4^+^ T Cell Responses in Cattle via *in vitro* Antigenic Expansion and Restimulation

**DOI:** 10.3389/fimmu.2020.01944

**Published:** 2020-09-02

**Authors:** Paola M. Boggiatto, Robert G. Schaut, Steven C. Olsen

**Affiliations:** ^1^Infectious Bacterial Diseases Research Unit, National Animal Disease Center, Agricultural Research Service, United States Department of Agriculture, Ames, IA, United States; ^2^Food Safety and Enteric Pathogens Research Unit, National Animal Disease Center, Agricultural Research Service, United States Department of Agriculture, Ames, IA, United States; ^3^Oak Ridge Institute for Science and Education (ORISE), ARS Participation Program, Oak Ridge, TN, United States

**Keywords:** *Brucella*, proliferation, IFN-γ, antigenic expansion, T cell responses

## Abstract

Bovine brucellosis, cause by infection with *Brucella abortus*, causes reproductive failure in cattle, has a major economic impact to producers, and as a zoonoses, it is a disease of public health concern. Characterization of the protective immune response against *Brucella* infection is important to our understanding of disease pathogenesis and for the development of diagnostic assays and vaccines. Most of the knowledge regarding protection against *Brucella* comes from studies in the murine model, but less is known about the immune responses in cattle. Assessment of antigen-specific T cell frequency and functional phenotype are critical to understand the immune status of the host, characterize mechanisms of protective immunity and immunopathology, and to predict immune protection. The frequency of circulating T cells specific for a particular pathogen is often very low, making analysis of such responses difficult. Our goal was to develop a flow-cytometry based approach to better track *Brucella*-specific T cell responses. Using peripheral blood mononuclear cells (PMBC) from *Brucella abortus* strain RB51-vaccinated cattle, we optimized an *in vitro* stimulation protocol based on a combination of antigen and pan-T cell stimulation. We then assessed RB51-specific T cell responses by concurrently measuring proliferation and cytokine production using flow-cytometry. This methodology enhances the detection of peripheral, *Brucella*-specific responses in cattle following RB51 vaccination. This protocol is versatile in that it can be modified to fit other *in vitro* stimulation systems and additional functional or phenotypic parameters can be added for flow cytometric detection and characterization of antigen-specific T cells.

## Introduction

*Brucella* species are facultative intracellular Gram-negative bacteria that infect multiple mammalian species. In its natural hosts, *Brucella* infection primarily results in reproductive failure and can result in major economic losses to producers. In addition, brucellosis is a zoonoses with great impact on public health worldwide. *Brucella abortus* primarily infects cattle and is the causative agent of bovine brucellosis. In the mouse model, the central role for IFN-γ-producing CD4^+^ T cells in protection against *Brucella* has been well-established (1–3). Yet, little is known regarding the protective immune responses in natural hosts. In cattle, vaccination with the commercial vaccine, *Brucella abortus* strain RB51, results in a proliferative T cell response (4, 5) that is correlated with protection from infection (6). Both CD4^+^ and CD8^+^ T cells contribute to this proliferative response (7) and assessment of the functional phenotype of antigen-specific responses demonstrated that CD4^+^ T cells are the major contributors of IFN-γ (7). Little else is known regarding the functional profile of these cells or the protective mechanisms of such responses.

The frequency of antigen-specific T cells is a critical determinant of immune efficacy (8), and therefore identification of such cells and their function are important for assessment of immune responses. Proliferation and cytokine production assays are commonly used to determine antigen-specific T cell responses to vaccines or infections. However, the frequency of circulating T cells specific for a particular antigen may be relatively low and undetectable by conventional assays. Additionally, when studying outbred populations, such as cattle, variability in these responses can make data interpretation difficult. *Brucella abortus* strains have been shown to have multiple immunodominant protein antigens (9), resulting in a multiclonal, heterogenous population of antigen-specific T cells, which may vary in their ability to proliferate and produce cytokines. However, as the immunodominant peptides have not been characterized, identification of *Brucella*-specific responses in *in vitro* assays are carried out using whole-killed antigen. Therefore, analyses of antigen-specific responses are done at the population level, where some less-dominant responses may be lost to background.

Proliferation and cytokine production can be discordant, especially if analyzing different T cell subpopulations such as effector or memory T cells. Central memory T cells (T_CM_) have a pronounced proliferation competence as compared to effector memory T cells (T_EM_), while T_EM_ can rapidly produce effector cytokines (8, 10). It has also been shown that some vaccination strategies can result in uncommitted T cells that respond to antigen via proliferation but do not produce effector cytokines (11). Additionally, proliferation and cytokine production are typically measured separately, disallowing for a thorough assessment of the functional potential of antigen-specific T cell responses.

To our knowledge, such a thorough analysis of functional capabilities has not been done for *Brucella-*specific responses. In order to accomplish this, we first optimized a flow cytometry-based assay to measure RB51-specific T cell proliferation and intracellular cytokine production following *in vitro* antigen stimulation of PMBCs. We then developed an *in vitro* recall response assay that includes a 2-step stimulation sequence utilizing both *Brucella*-specific and pan-T cell stimulation followed by concurrent assessment of T cell proliferation and intracellular cytokine production. The idea behind this assay was to first take advantage of antigen expansion to increase the frequency of antigen-specific T cells followed by a activation step with phorbol 12-myristate 13-acetate (PMA) and a calcium ionophore, ionomycin, to increase cytokine production (12, 13). Using this approach, we are able to enhance detection of *Brucella*-specific T responses and begin to further characterize the functional potential of the response.

## Materials and Methods

### Animal Vaccination

Hereford heifers, ~6–8 months old, were housed outdoors on the National Animal Disease Center (NADC) campus in Ames, Iowa. Following a 3-month acclimation period, heifers were divided into control and vaccine groups. Control animals (*n* = 12) were left unvaccinated, while the vaccine group (*n* = 18) was immunized with 3 × 10^10^ colony forming units (CFU) of *Brucella abortus* strain RB51 vaccine (Lot number 3180; Colorado Serum Company, Denver, CO) via the intramuscular route in the cervical region. Blood samples from all animals were collected via venipuncture of the jugular vein in order to assess peripheral immune responses to vaccination. The data shown here are representative of blood collected at 16 weeks post-vaccination. All work involving animals was conducted with the approval of the NADC institutional animal care and use committee (IACUC, protocol number ARS-2017-690).

### Isolation of Peripheral Blood Mononuclear Cells (PBMC)

PBMC were isolated from whole blood via density gradient centrifugation. Briefly, 30 ml whole blood were collected into tubes containing acid-citrate dextrose (ACD). The blood was centrifuged at 1,200x g for 30 min at room temperature (RT). The white blood cell layer was collected and diluted 1:2 with culture grade, Dulbecco's phosphate-buffered saline (DPBS) (Gibco, Thermo Fisher, Waltham, MA) and centrifuged again at 1,200x g for 30 min. at RT. The buffy coat was again collected and layered onto a Ficoll gradient (1.077 g/ml) (Sigma-Aldrich, St. Louis, MO) and centrifuged at 1,200x g for 30 min at RT. PBMCs were then harvested and washed in DPBS by centrifugation at 300x g for 10 min at RT. PBMC were filtered through a 40 μm strainer, live cell number was determined via trypan blue exclusion, and resuspended to a final concentration of 10 × 10^6^ cells per ml in complete RPMI 1640 (cRPMI) (Gibco Life Tech, Thermo Fisher Scientific) media consisting of 20% heat-inactivated fetal bovine serum (FBS) (HyClone™ Cytiva, Marlborough, MA), 100 U/ml penicillin, 100 μg/ml streptomycin, 2 nM glutamine, 1% sodium pyruvate, 1% non-essential amino acids, 1% essential amino acids (Sigma Life Science, St. Louis, MO), 50 μM 2-beta mercaptoethanol (Sigma Aldrich), and 1% HEPES buffer (Gibco Life Tech, Thermo Fisher Scientific).

### PBMC Labeling for Proliferation

PBMC were labeled using the CellTrace® violet proliferation kit (Invitrogen, Thermo Fisher Scientific), according to manufacturer's recommendations with some modifications. Briefly, ~30 × 10^6^ cells were washed in DPBS via centrifugation at 300x g for 10 min RT. Supernatant was discarded and cells resuspended in 100 μl of CellTrace® suspension (5 μM working stock) per 10 × 10^6^ cells, vortexed, and incubated for 20 min at RT with periodic vortexing. Cells were then washed in 10 ml of DPBS via centrifugation at 300x g for 10 min at RT, and resuspended in cRPMI media to a concentration of 10 × 10^6^ cells/ml. Adequate CellTrace® staining for each sample was assessed via flow cytometry using BD FACSymphony™ A5 flow cytometer (BD Bioscience, San Jose, CA). Data was analyzed using FlowJo® (Tree Star, Inc. San Diego, CA).

### *In vitro* RB51 Recall Response Assay

For *in vitro* recall responses, 100 μl of cell suspension was the plated onto 96-well flat-bottom plates and stimulated with 100 μl of γ-irradiated RB51 (1 × 10^7^ CFU/well), 100 μl (0.5 μg/well) of Concavalin A (Sigma-Aldrich), or 100 μl of media only. All conditions were plated in triplicate and plates were incubated at 37°C with 5% CO_2_ for 7 days. To measure intracellular cytokine production, some wells were treated with a 1x solution of eBioscience™ Protein transport inhibitor (500x Brefeldin A) (Thermofisher Scientific) overnight for 16 h.

### Two-Step *in vitro* Stimulation Assay: RB51 Antigenic Expansion and Restimulation

For the first step in stimulation (antigenic expansion), PBMC were stimulated with RB51 antigen as described above, and incubated at 37°C with 5% CO_2_ for 7 days. For the second step of stimulation (restimulation), each condition was treated with either a 1x solution of eBioscience™ Cell Stimulation and Protein Transport Inhibitor cocktail (500x phorbol 12-myristate 13-acetate (PMA), ionomycin, brefeldin A, and monensin), a 1x solution of eBioscience™ Protein Transport Inhibitor cocktail (500x Brefeldin A and monensin), or a 1x solution of eBioscience™ Cell Stimulation cocktail (500x phorbol 12-myristate 13-acetate (PMA) and ionomycin) (Thermo Fisher Scientific) overnight for 16 h prior to harvesting at day 7.

### Cell Surface Receptor and Intracellular Cytokine Staining

At the day 7 harvest PBMCs were washed twice with DPBS via centrifugation at 300x g for 5 min at RT. Cells were incubated with a fixable viability dye (eBioscience™, Thermo Fisher Scientific) for 20 min at 4°C, and then washed once in DPBS and once in FACS buffer (PBS with 0.5% FBS) via centrifugation at 300x g for 5 min at RT. Cells were incubated for 15 min at RT with a primary mouse anti-bovine γδ antibody (IgG2b, TCR1-N24; Washington State University, Pullman, WA) followed by incubation with an anti-mouse IgG2b BUV395-labeled antibody (BD Biosciences) for 15 min at RT. Cells were then washed twice in FACS buffer and incubated with a FITC-labeled anti-bovine CD4 (CC8) and APC-labeled anti-bovine CD8 (CC63) (Bio-Rad, Hercules, CA) antibodies for 15 min at RT. Following incubation, cells were washed twice in FACS buffer, and then fixed and permeabilized using the BD Cytofix/Cytoperm™ kit (BD Biosciences), according to manufacturer's recommendations. Cells were then incubated with a PE-labeled anti-bovine IFN-γ (CC302) in 1x Wash/Perm buffer for 30 min at RT, and then washed once with 1x Wash/Perm buffer and once in FACS buffer. Cells were resuspended in 200 μl of FACS buffer and analyzed using a BD FACSymphony™ A5 flow cytometer (BD Biosciences). Data was analyzed using FlowJo® software (Tree Star, Inc.).

### Statistical Analysis

All statistical analyses were performed using GraphPad Prism 8 (GraphPad Software, San Diego, CA). For pair-wise comparisons, *t*-tests were performed correcting for multiple comparisons using the Bonferroni-Dunn method. Correlation analysis were computed using Pearson correlation coefficients. *P* values of < 0.05 were considered statistically significant.

## Results

### RB51-specific Proliferative T Cell Responses

**Figure 1:**
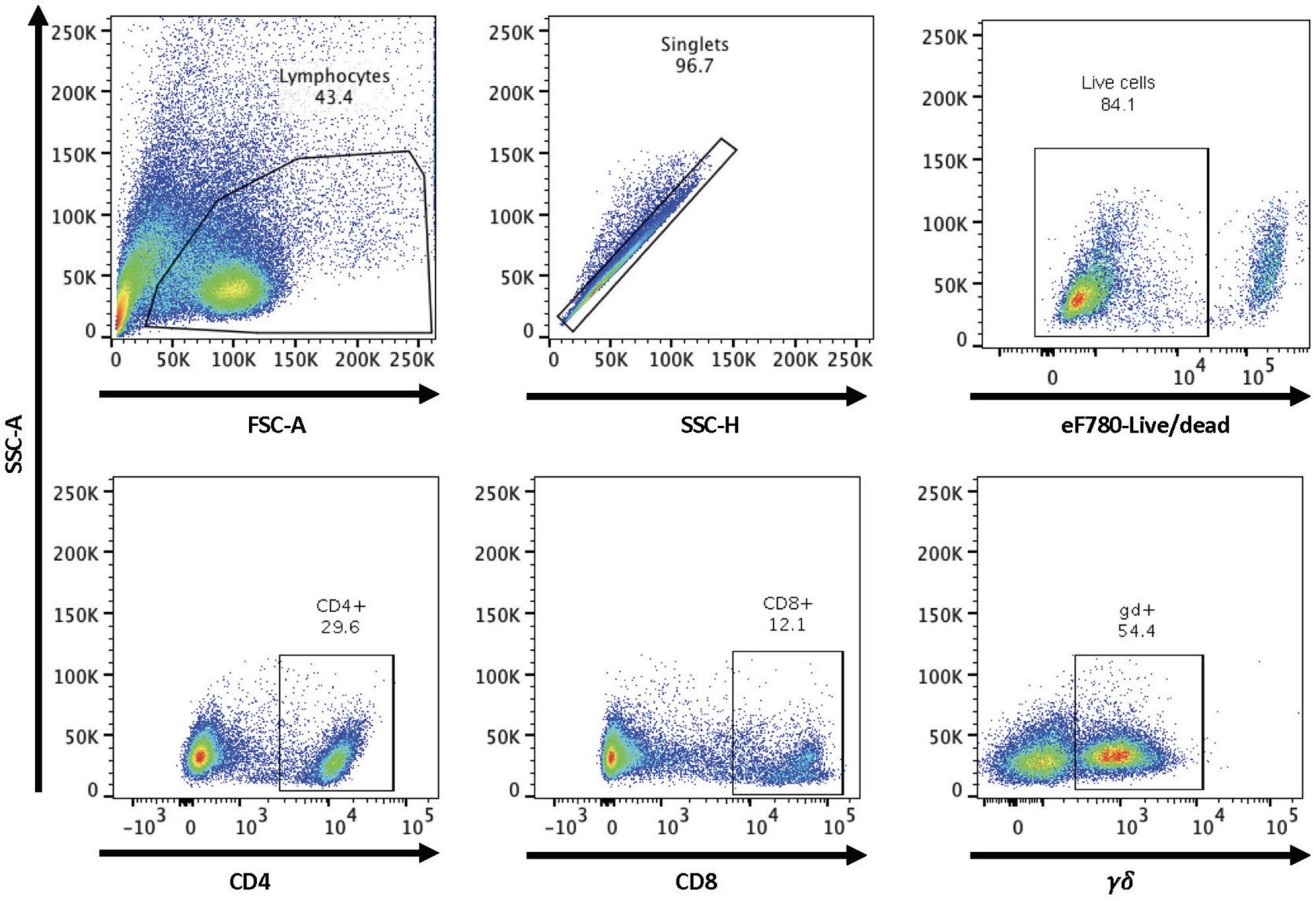
Gating strategy for flow cytometry analysis. Shown are representative dot plots showing the gating strategy for lymphocytes, singlets, live/dead determination, and CD4, CD8, and γδ T cell subsets.

**Figure 2:**
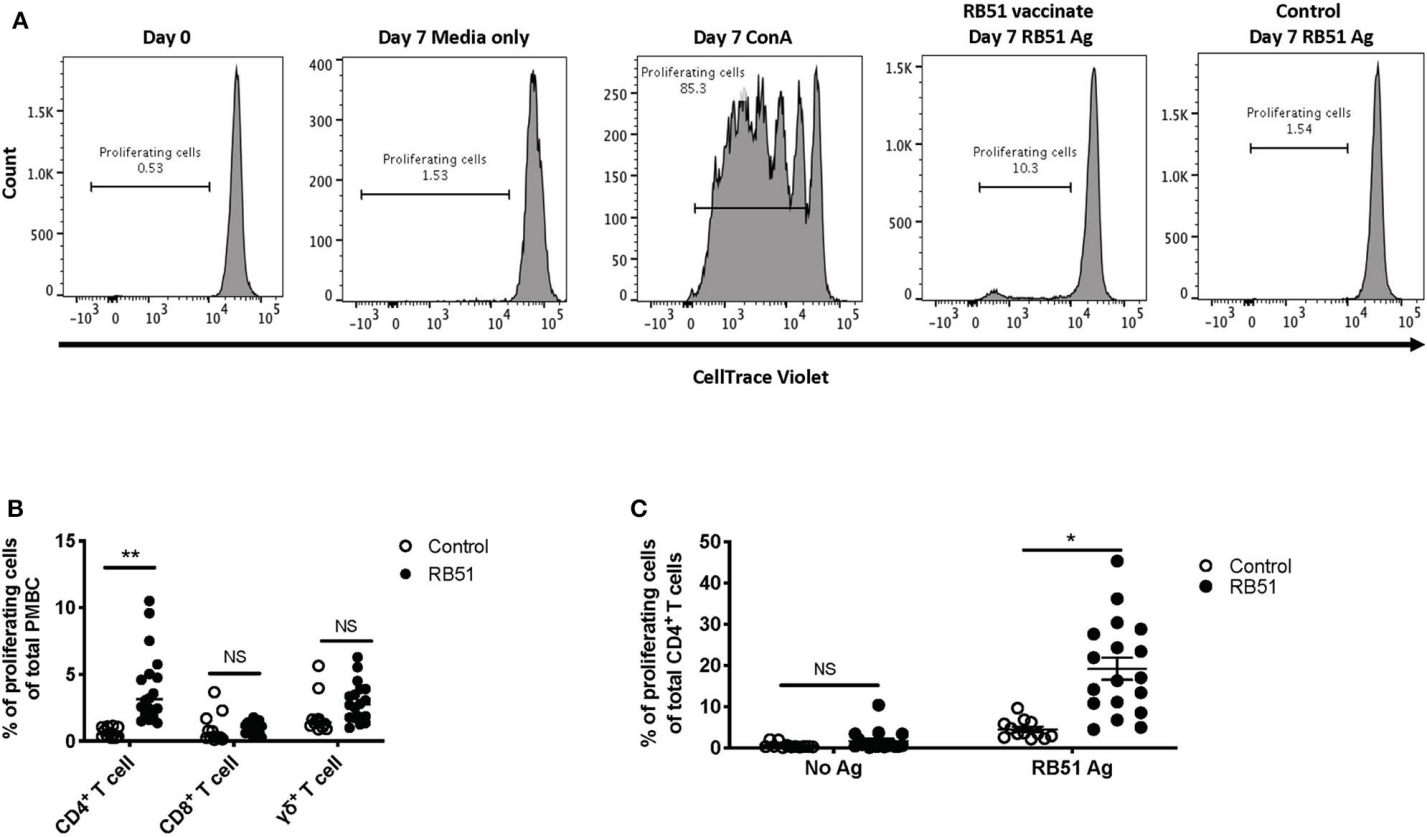
RB51-specific proliferation following *in vitro* antigen stimulation of PBMC from control and RB51-vaccinated animals. Representative histograms showing PBMC CellTrace™ violet staining of total PBMC prior to culture, and following 7 days of culture in media only, with ConA, and RB51 antigen **(A)**. Frequency of proliferating PBMC from control (open circles) and vaccinated animals (black circles) following a 7-day, *in vitro* RB51 antigen stimulation, broken down by T cell subset including CD4^+^, CD8^+^ and γδ T cells **(B)**. Frequency of proliferating CD4^+^ T cells from control and vaccinated animals left unstimulated or stimulated with RB51 antigen *in vitro* **(C)**. Individual animals with mean and standard deviations from the mean shown for all graphs. *indicates *P* ≤ 0.05, **indicates *P* ≤ 0.001, and NS indicates no statistical significance.

In order to assess RB51-specific proliferative responses, we first optimized a flow cytometry-based assay using an intracellular dye to detect proliferation via dye dilution. The gating strategy for all flow cytometry analysis is shown in Figure 1. Following labeling with CellTrace® violet dye, all samples were checked to ensure uniform staining prior to plating (Figure 2A, first panel). PBMC left unstimulated (medium only) did not proliferate during the 7-day incubation period, while cells stimulated with ConA demonstrated several rounds of proliferation in response to mitogen (Figure 2A, second and third panels). PBMC from vaccinated animals, but not control animals, proliferated in response to RB51 antigen stimulation (Figure 2A, fourth and fifth panels, and Figure 2B). When we analyzed the specific T cell subsets contributing to this proliferative response, we found it to be predominantly composed of CD4^+^ T cells (Figure 2B). When comparing the RB51-specific proliferation of PBMC from controls and vaccinates, we only observe a statistically-significant difference (*P* = 0.0002) within the CD4^+^ T cell subset, whereas no such differences were observed for CD8^+^ or γδ T cells between control and vaccinated animals (Figure 2B) at the timepoint analyzed. When stimulated with ConA, CD4^+^, CD8^+^ and γδ T cells undergo several rounds of proliferation, suggesting that the differences in proliferation to antigen stimulation are not related to an inability of the different subsets to proliferate (Supplementary Figure 1).

When assessing the CD4^+^ T cell response following stimulation, ~19% of CD4^+^ T cells proliferate in response to RB51 antigen. This proliferation is antigen-specific as it is significantly different (*P* = 0.025) when compared to control animals (Figure 2C). Altogether, the data shows that this proliferation assay allows us to detect RB51-specific proliferative responses and distinguish the T cell subsets contributing to this response.

### RB51-specific IFN-γ T Cell Responses

**Figure 3:**
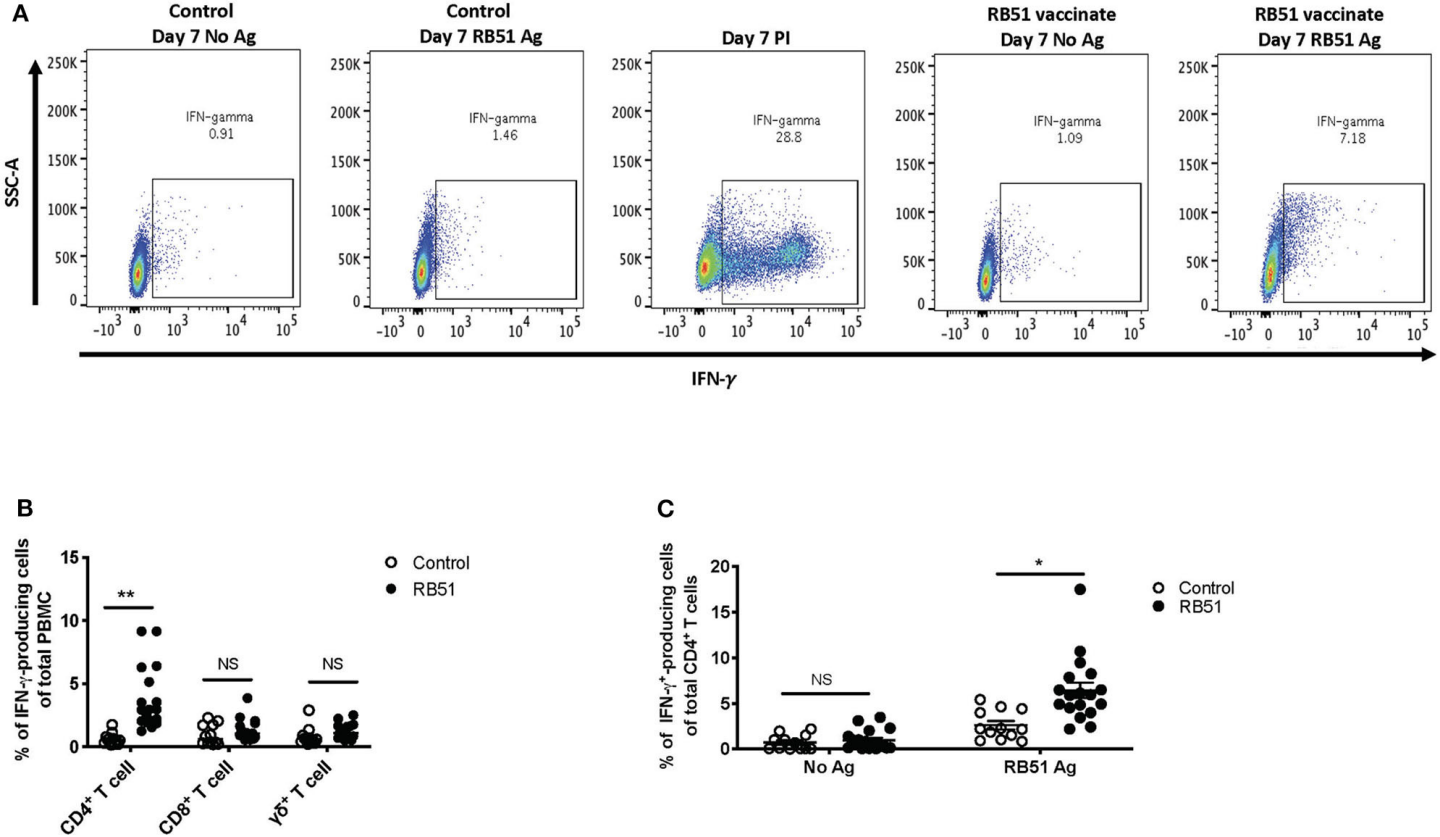
RB51-specific IFN-γ production following *in vitro* stimulation of PBMC from control and RB51-vaccinated animals. Shown are representative side scatter (SSC-A) and IFN-γ dot plots of PBMC from control and RB51 vaccinated animals following at 7-day culture left unstimulated or stimulated with RB51 antigen or with PMA & ionomycin (PI) **(A)**. Shown are frequencies of IFN-γ-producing PMBC from control (open circles) or vaccinated animals (black circles), broken down by T cell subsets including CD4^+^, CD8^+^, and γδ T cells **(B)**. Frequency of IFN-γ-producing CD4^+^ T cells from control and vaccinated animals left unstimulated or following stimulation with RB51 antigen **(C)**. Individual animals with mean and standard deviations from the mean shown for all graphs. *indicates *P* ≤ 0.05, **indicates *P* ≤ 0.001, and NS indicates no statistical significance.

*In vitro* recall responses, as described above, were performed and IFN-γ production was assessed via flow cytometry using intracellular cytokine staining. In the absence of antigen stimulation, we observed ~1% of IFN-γ-producing cells from total PBMC from control or vaccinated animals (Figure 3A, first and fourth panels), and ~30% of IFN-γ-producing cells when stimulated with PMA and ionomycin (PI) only, as a positive control (Figure 3A, third panel). When PBMCs were stimulated with RB51 antigen, we observed a higher frequency of IFN-γ-producing PBMCs from vaccinated animals as compared to control animals (Figure 3A, third and fourth panels). Similar to the proliferation data, as compared to control animals, statistically significant different (*P* = 0.0025) frequencies of IFN-γ-positive cells were only observed within the CD4^+^ T cell subset of vaccinated animals (Figure 3B). The frequency of IFN-γ-producing CD8^+^ or γδ T cells were not statistically different (*P* = 0.24 and 0.08, respectively) between control and vaccinated animals (Figure 3B). Closer analysis of the CD4^+^ T cell population shows that an average of 6.4% of CD4^+^ T cells produce IFN-γ in response to RB51 antigen. This response is antigen-specific, as we do not observe differences (*P* = 0.57) in the frequency of IFN-γ-producing CD4^+^ T cells between controls and vaccines in the absence of antigen stimulation (Figure 3C). These data demonstrate that this assay is able to detect RB51-specific IFN-γ production from PBMC cultures and these results are consistent with previous reports indicating IFN-γ is primarily produced by CD4^+^ T cells following RB51 vaccination (14).

### Lack of Correlation Between Proliferation and IFN-γ Production

**Figure 4:**
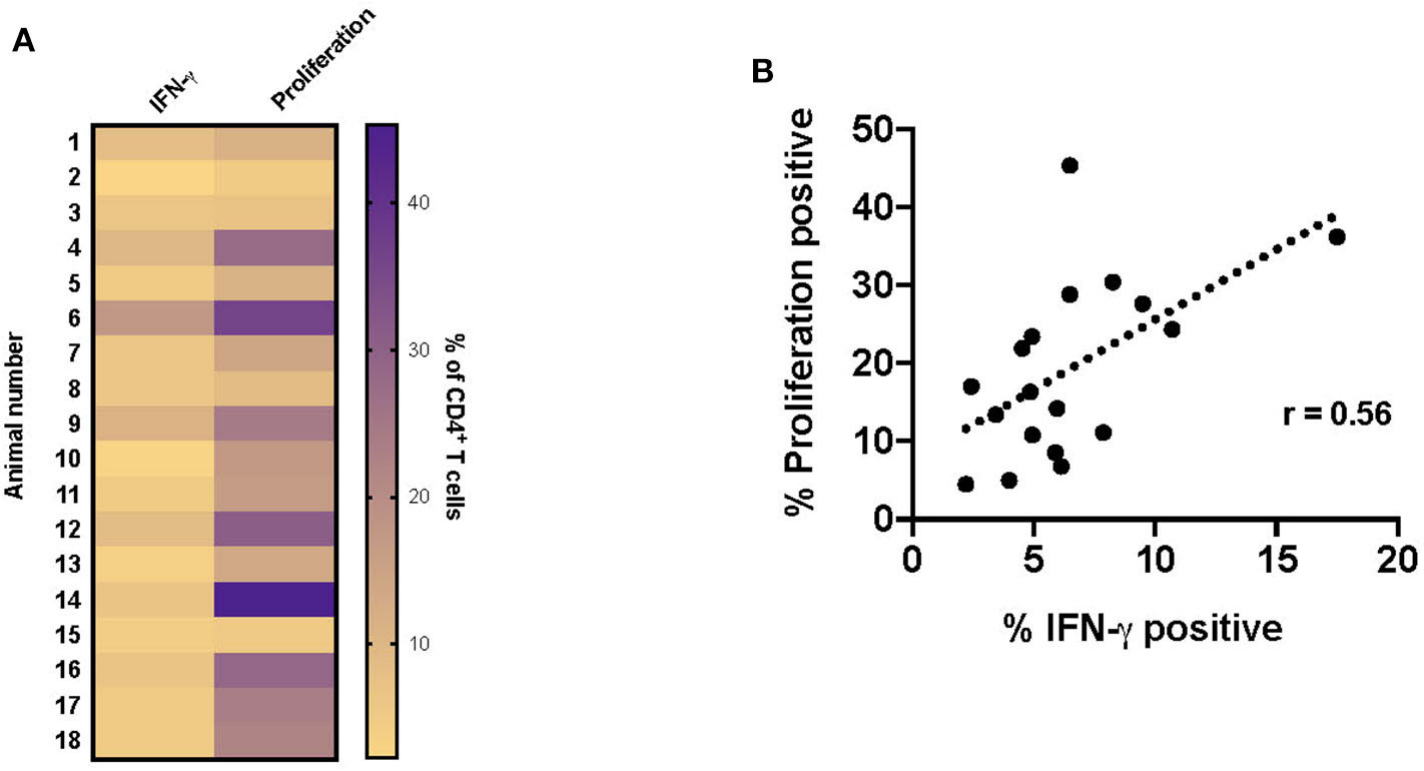
Weak correlation between CD4^+^ T cell proliferation and IFN-γ production following RB51 antigen stimulation of PBMC from vaccinated animals. Heat map of the frequency of proliferating and IFN-γ producing CD4^+^ T cells using color scale **(A)**. Higher frequency values are indicated by purple, while lower frequency values are indicated by gold, numbers indicate individual animals. Pearson correlation analysis of the frequency of IFN-γ-producing and proliferating CD4^+^ T cells in response to RB51 antigen stimulation, showing poor correlation between the two function **(B)**.

Since the majority of the proliferative and IFN-γ response observed at this timepoint was associated with CD4^+^ T cells, the data presented in the remainder of the study primarily focuses on this subset. Overall, both proliferation and IFN-γ responses are increased in vaccinated animals in response to RB51 antigen. However, proliferative responses tended to predominate, in other words, a higher frequency of CD4^+^ T cells proliferate in response to antigen than make IFN-γ (Figure 4A). Furthermore, and not surprisingly, there is a weak correlation (*r* = 0.56; CI = 0.13–0.81; *P* = 0.015) between the frequency of proliferating and the frequency of IFN-γ-producing CD4^+^ T cells (Figure 4B). However, because these functional responses are typically assessed separately, it is difficult to make specific conclusions regarding the functional capacity of each cell. Therefore, we sought to explore the relationship between proliferation and cytokine production in response to RB51 antigen by combining these two assays and assessing both functional phenotypes concurrently.

**Figure 5:**
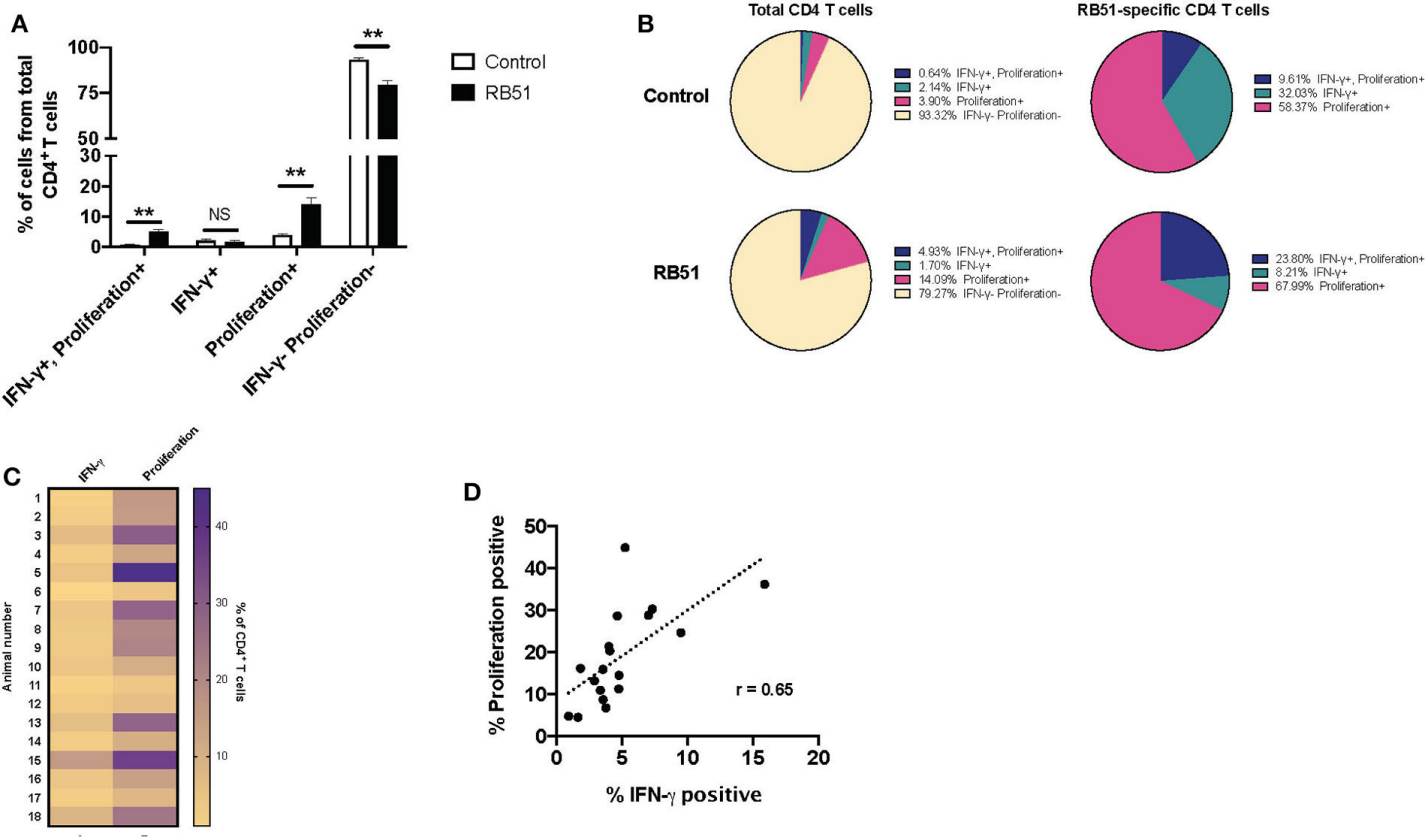
Proliferative responses to RB51 antigen predominate within responding CD4^+^ T cells following antigen stimulation of PMBC from RB51-vaccinated animals. Bar graph showing frequency of IFN-γ^+^/proliferation^+^, IFN-γ^+^, proliferation^+^, or IFN-γ^−^/proliferation^−^ CD4^+^ T cells from control (open square) and vaccinated animals (black squares) following 7-day *in vitro* RB51 antigen stimulation **(A)**. Pie charts showing the breakdown of specific function based on IFN-γ production, proliferation, and their combinations, for total CD4^+^ T cells (first two circles) and for RB51-responding CD4^+^ T cells for control and vaccinated animals **(B)**. Heat map of the frequency of proliferating and IFN-γ producing CD4^+^ T cells using color scale **(C)**. Higher frequency values are indicated by purple, while lower frequency values are indicated by gold, numbers indicate individual animals. Pearson correlation analysis of the frequency of IFN-γ-producing and proliferating CD4^+^ T cells in response to RB51 antigen stimulation, showing poor correlation between the two functions **(D)**. Mean and standard deviations from the mean are shown. **indicates *P* ≤ 0.001, and NS indicates no statistical significance.

PMBC were stimulated as before and proliferation and IFN-γ production from CD4^+^ T cells were analyzed concurrently (Supplementary Figure 2A). We observed significant differences between control and vaccinated animals in the frequency of CD4^+^ T cells that proliferated and in the frequency of CD4^+^ T cells that proliferated and produced IFN-γ (Figure 5A). In control animals, we observed a small frequency, ~6.7%, of CD4^+^ T cells responding to stimulation. Of these cells, 58.37% only proliferated, 32% only produced IFN-γ, and 9.6% proliferated and produced IFN-γ (Figure 5B, top pie charts). In contrast, in vaccinated animals, we observed that ~20% of CD4^+^ T cells responded to antigen stimulation.

Interestingly, of the total CD4^+^ T cell population from vaccinated animals, 14.0% of cells only proliferated, 1.7% of cells only produced IFN-γ, and 4.93% of cells proliferated and produced IFN-γ (Figure 5B, bottom pie charts). When we look more closely at the population of cells responding to antigen (i.e., cells proliferating and/or producing IFN-γ), we observe that 67.99% of cells only proliferate, 8.21% only produce IFN-γ, and 23.8% proliferate and produce IFN-γ (Figure 5B). By combining these two functional assays, we show that RB51 antigen-specific CD4^+^ T cells are not all the same, as some cells only proliferate, some only produce cytokine, and some are able to perform both functions.

Concurrent assessment of proliferation and IFN-γ production allowed us to more accurately evaluate the relationship between these two functional parameters. Consistent with previous findings, proliferation of CD4^+^ T cell predominates as compared to IFN-γ production in response to RB51 antigen stimulation (Figure 5C), and there is a weak correlation between these two functional parameters (Figure 5D). Altogether, these data demonstrate that proliferation may be a more sensitive parameter for the assessment of antigen-specific responses following RB51 vaccination. However, the data also demonstrates that the responding population of CD4^+^ T cells may vary in their functional capacities.

### Enhancing IFN-γ Production From RB51-specific CD4^+^ T Cells

**Figure 6:**
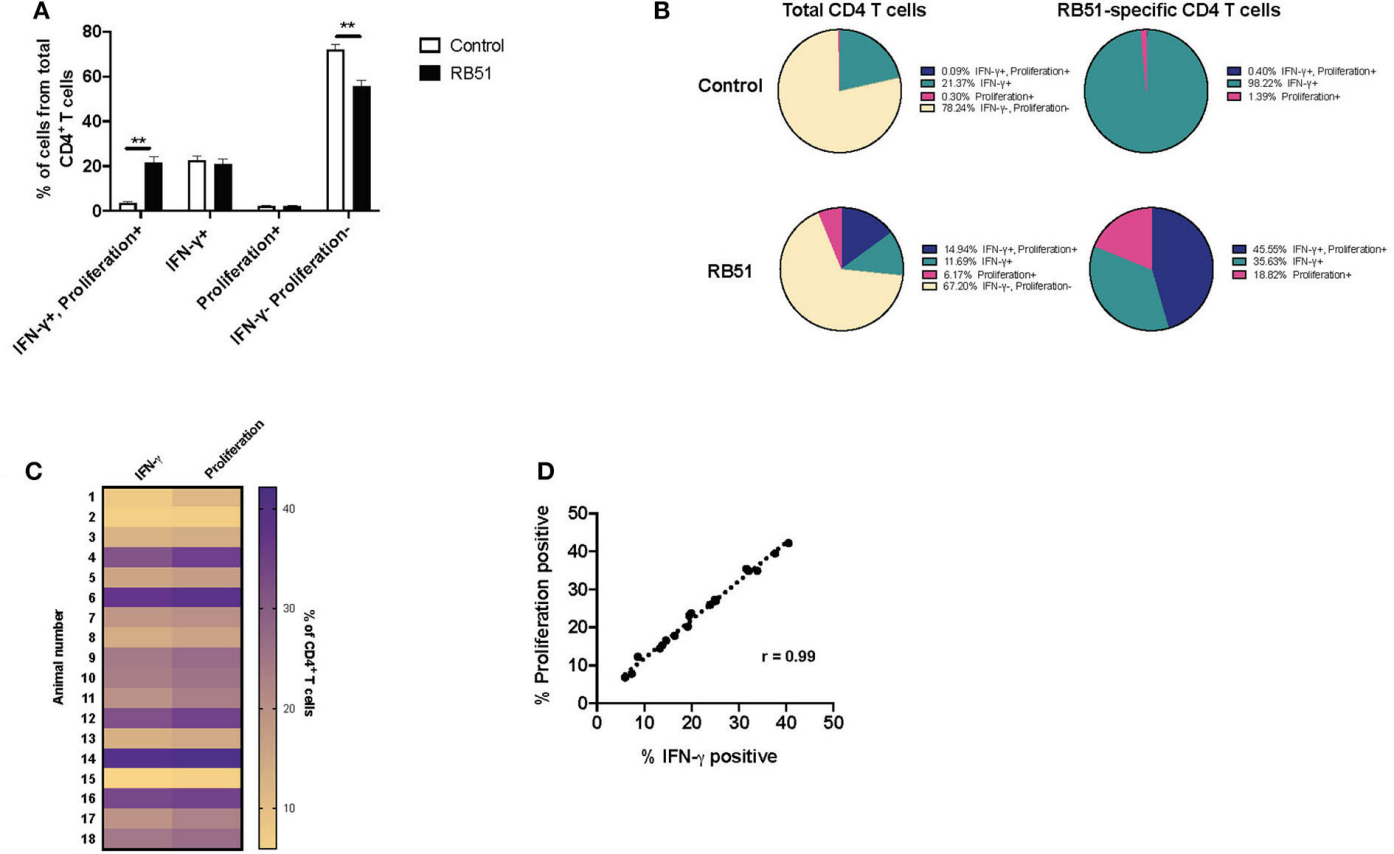
Enhanced and honed detection of functional profiles RB51-specific CD4^+^ T cell responses. Bar graph showing frequency of IFN-γ^+^/proliferation^+^, IFN-γ^+^, proliferation^+^, or IFN-γ^−^/proliferation^−^ CD4^+^ T cells from control (open square) and vaccinated animals (black squares) following 7-day *in vitro* RB51 antigen stimulation followed by restimulation with PMA & Ionomycin (PI) **(A)**. Pie charts showing the breakdown of specific function based on IFN-γ production, proliferation, and their combinations, for total CD4^+^ T cells (first two circles) and for RB51-responding CD4^+^ T cells (second two circles) for control (top) and vaccinated (bottom) animals **(B)**. Heat map of the frequency of proliferating and IFN-γ producing CD4^+^ T cells using color scale **(C)**. Higher frequency values are indicated by purple, while lower frequency values are indicated by gold, numbers indicate individual animals. Pearson correlation analysis of the frequency of IFN-γ-producing and proliferating CD4^+^ T cells in response to RB51 antigen stimulation, showing a strong correlation between the two functions **(D)**. Mean and standard deviations from the mean are shown. **indicates *P* ≤ 0.001, and NS indicates no statistical significance.

Overall, the RB51-specific CD4^+^ T cell response appears to be comprised primarily of proliferating cells (>90%), while a smaller percentage of the response is composed of IFN-γ production. We wondered if we could enhance cytokine production from these cells. In order to test this, we added a second step of stimulation to our *in vitro* assay. For the first 7 days of culture, cells were stimulated with RB51 antigen or left unstimulated, and for the last 16 h, PI, were added in order to induce cytokine production. As above, proliferation and IFN-γ production were then assessed concurrently (Supplementary Figures 2A,B). Not surprisingly, the addition of PI changed the functional prolife of CD4^+^ T cells toward an increased frequency of IFN-γ-producing cells in both control and vaccinated animals (Figures 6A,B), as compared to the functional profile of cells only stimulated with RB51 antigen (Figures 5A,B). As before, we observed statistically significant differences in the frequency of CD4^+^ T cells that proliferate and produce IFN-γ in response to RB51 antigen stimulation between control and vaccinated animals (Figure 6A). In control animals, ~21% of CD4^+^ T cells either proliferate or produce IFN-γ following *in vitro* culture, however, of these cells >98% are IFN-γ-producers only, which can be attributed to the addition of PI (Figure 6B). In contrast, with vaccinated animals, we see that ~32% of CD4^+^ T cells respond to antigen stimulation. Of these cells, 45.5% proliferate and produce IFN-γ, 35.6% only produce IFN-γ, and 18.82% only proliferate in response to RB51 antigen stimulation and restimulation with PI (Figure 6B). With the addition of PI, IFN-γ production by antigen-specific cells (i.e., proliferating CD4^+^ T cells) is increased, and we find that the magnitude of the proliferative and cytokine responses is now similar (Figure 6C). Additionally, we observed a strong correlation between proliferation and cytokine production in response to RB51 antigen stimulation (Figure 6D). These data demonstrate that RB51-specific CD4^+^ T cells are capable of producing IFN-γ if the stimulus is provided.

### Restimulation Allows for Honed Detection of RB51-specific CD4^+^ T Cells From Vaccinated Animals

**Figure 7:**
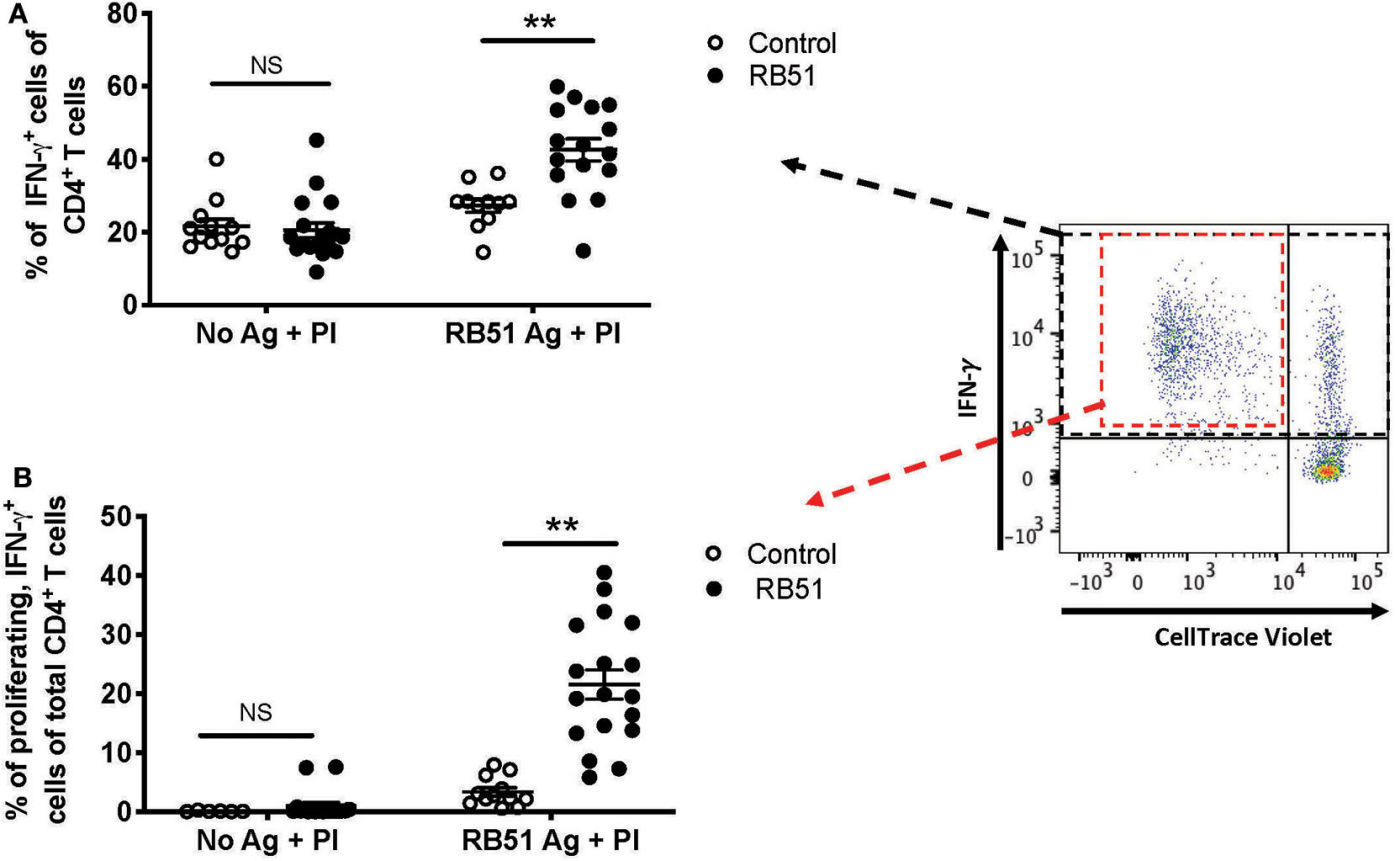
Restimulation with PMA/Ionomycin (PI) following RB51 antigen stimulation results in enhanced and honed detection of RB51-specific CD4^+^ T cell responses. Frequency of IFN-γ-producing CD4^+^ T cells following a 7-day *in vitro* culture of PMBC with or without RB51 antigen stimulation and restimulated with PMA & ionomycin (PI) 16 h prior to harvest, from control (open circles) or RB51-vaccinated (black circles) animals **(A)**. Shown are the frequency of CD4^+^ T cells that are both proliferating and producing IFN-γ with or without RB51 antigen stimulation followed by PI re-stimulation **(B)**. Individual animals with mean and standard deviations from the mean are shown for both graphs. **indicates *P* ≤ 0.001, and NS indicates no statistical significance.

Enhancing the detection of RB51-specific CD4^+^ T cells facilitates the identification of vaccinated animals. Using the 2-step *in vitro* stimulation system described above, we wanted to determine if we could more clearly distinguish vaccinated from control animals. When we analyzed the frequency of CD4^+^ T cells that only produced IFN-γ without RB51 antigen stimulation, addition of PI resulted in similarly (*P* = 0.72) robust IFN-γ production by CD4^+^ T cells, from both control and vaccinated animals (Figure 7A). However, when PMBC were first stimulated with RB51 antigen and then PI, we observe a statistically-significant increase (*P* = 0.0006) in the frequency of IFN-γ-producing CD4^+^ T cells in vaccinated animals as compared to controls (Figure 7A). Not surprisingly, stimulation with PI prior to harvest enhances the detection of antigen-specific IFN-γ-producing cells, therefore making the identification of such responses easier. Interestingly, while IFN-γ responses are enhanced in both control and vaccinated animals, we can still detect statistically-significant differences between control and vaccinated animals as long as antigen stimulation occurs first.

The addition of PI to the cultures triggers IFN-γ production from any activated T cell, regardless of antigen specificity. However, PI would have little to no effect on proliferation of cells in the short stimulation time, and therefore, any proliferation observed would be RB51-specific. We postulated that by gating specifically on the population of CD4^+^ T cells that are both proliferating and producing IFN-γ in response to RB51 antigen, we would be able to enhance the distinction between vaccinated and control animals. In the absence of prior RB51 antigen stimulation, the average frequency of proliferating, IFN-γ-producing CD4^+^ T cells was <1% for both control and vaccinated animals, even after PI stimulation (Figure 7B). However, when cells are first stimulated with RB51 antigen, we observed a robust and significant increase (*P* = 0.00002) in the frequency of proliferating, IFN-γ-producing CD4^+^ T cells from vaccinated animals as compared to controls (Figure 7B). The concurrent assessment of proliferation and cytokine production following the 2-step stimulation assay enhances our ability to detect RB51-specific responses.

## Discussion

Most of our knowledge regarding protective immunity following infection or vaccination with *Brucella abortus* comes from experiments performed in mice. We extrapolate this information into cattle, but there is a limited understanding of the protective immune response elicited within this natural host. A broader understanding of the immune mechanisms that confer protection to cattle will aid in the development and evaluation of new immunological approaches to detect and control brucellosis. Some factors limiting the study of *Brucella* responses in cattle include costs associated with the research, appropriate housing facilities, reagent availability and the limited immune responses observed in peripheral blood.

The low frequency of antigen-specific T cells following certain infections or immunizations poses a problem to the understanding of protective immunity. In the work presented here, we sought to develop and optimize an *in vitro* assay in order to enhance the detection of *Brucella*-specific T cell responses in cattle following RB51 vaccination. In this assay, we take advantage of their proliferative potential to increase the frequency of RB51-specific cells via antigen stimulation (i.e., antigenic expansion), followed by a short period of stimulation with PI to stimulate cytokine production. Additionally, we combine assessment of proliferation and intracellular cytokine staining to more closely analyze the functional potential of RB51-specific CD4^+^ T cells.

Similar approaches have been previously described in order to enhance detection of cytokine responses from human PBMC following immunizations (12, 13). One potential caveat to this approach is the bystander effect, or proliferation by non-RB51-specific cells driven by cytokines produced by proliferating cells (15), that could partially account for the overall T cell population after antigen stimulation. However, in our assay, while we observe CD4^+^ T cell proliferation following RB51 antigen stimulation of vaccinated animals, we do not see a significant increase in the proliferation of CD8^+^ or γδ T cells in these cultures (Figure 2B). These data would suggest that if there is a bystander effect, in our system, it is rather minimal. Accordingly, others have shown that even with the addition of exogenous proliferative cytokines, such as interleukin (IL)-2, low backgrounds of non-specific responses can be maintained (13). Nevertheless, this phenomenon should be considered when utilizing *in vitro* antigenic expansion as a readout for immune responses.

Bovine T cell responses to vaccination with RB51 have been extensively studied, primarily through proliferation [(4–6)], but not until recently, has there been an analysis of specific T cell subset proliferation (7, 14). Additionally, there is limited information regarding cytokine profiles, but IFN-γ production appears to be the primary cytokine produced by CD4^+^ T cells (14). When we assessed RB51-specific proliferation and cytokine production, we observed a poor correlation between these two functional phenotypes and found that the proliferative response is greater than the IFN-γ response (Figure 4). By combining these two assays, we can more closely assess the functional capacity of RB51-specific T cell responses. Proliferation and cytokine production are triggered by antigen stimulation, yet proliferation does not always equal effector cytokine production. Antigen-specific cells that only produce IL-2 (i.e., able to proliferate) and that do not secrete IFN-γ have been previously described (11, 16, 17). Such cells can represent different T cell subsets. For example, protein vaccines have been shown to induce uncommitted, IL-2-producing T cells that can differentiate into effectors cells following antigen stimulation (11). Alternatively, IL-2-producing cells could represent different populations of memory cells. Central memory T cells (T_CM_) are able to primarily proliferate in response to antigen stimulation, while effector memory T cells (T_EM_) only produce cytokines (8, 10). It should also be noted that differences in proliferation vs. cytokine production potential could also be tied to the nature antigenic stimulus. The T cell receptor signaling pathways that trigger these events are distinct and can be uncoupled from one another depending on signal strength (i.e., strong vs. weak peptides) (18).

While here we did not measure IL-2 directly, we are able to see a population of cells that only proliferate in response to RB51 antigen, but do not produce IFN-γ (Figure 5). Interestingly, these CD4^+^ T cells are capable of producing IFN-γ when stimulated with PI (Figure 6), suggesting the functional potential is there. With this assay, we can now begin to dissect the overall cytokine-production profile of RB51-specific T cells, which may have been previously undetectable. Functional distinction of T cells in terms of effector or memory T cells population following RB51 vaccination, or *Brucella* infection, is an area that remains unexplored. T cell responses following RB51 vaccination take time to develop, with peak proliferative responses occurring at ~10–12 weeks post-vaccination (6). Currently, further work is underway in our laboratory to assess these responses using the assay presented in this manuscript at different time points following vaccination. Taking this approach, we can look more closely at the functional profile of responding cells and also assess cytokines in addition to IFN-γ to gain further understanding into the immune components that lead to protection. To our knowledge, this is the first time that this identification of functional differences in RB51-specific CD4^+^ T cells has been made.

At the timepoint used for this analysis, we did not see a significant proliferative or IFN-γ response from CD8^+^ or γδ T cells, and therefore focused on the CD4^+^ T cell response. However, it should be noted that this protocol can be applied to CD8^+^ and γδ T cells (Supplementary Figures 3, 4). Significant proliferation of CD8^+^ T cells has been observed by others (14), and while we did not here, this could be attributed to the timepoint used for this analysis. The function of γδ T cells following RB51 vaccination remains poorly characterized. However, this assay can be applied to also enhance the detection of these T cell subsets and further characterize their functional phenotype following vaccination.

The assay presented here can be adapted to any infectious and non-infectious model that requires measurement of T cell responses. The information gathered can provide useful information as to the functional potential of antigen-specific T cell responses that were not measurable by traditional means. To our knowledge, this is the first time that such an assay has been used in cattle to characterize *Brucella*-specific responses. As we gain insight into the protective immune responses in cattle against *Brucella* we can develop assays that are better suited for diagnostic purposes and for determination of vaccine efficacy.

## Data Availability Statement

All datasets generated for this study are included in the article/Supplementary Material.

## Ethics Statement

The animal study was reviewed and approved by National Animal Disease Center Institutional Animal Use and Care Committee (IACUC).

## Author Contributions

PB designed and performed the experiments, analyzed and interpreted the data, and wrote the manuscript. RS designed experiments, analyzed and interpreted data, and edited the manuscript. SO analyzed and interpreted the data and edited the manuscript. All authors contributed to the article and approved the submitted version.

## Conflict of Interest

The authors declare that the research was conducted in the absence of any commercial or financial relationships that could be construed as a potential conflict of interest.

## Supplementary Material

Supplementary Figure 1 T cell subset proliferation in response to ConA stimulation. Representative histograms of CellTrace™ violet dilution for PBMC gated on CD4-, CD8- and γδ-positive cells.

Supplementary Figure 2 Proliferative and IFN-γ responses from RB51-specific CD4^+^ T cells following *in vitro* antigen stimulation. Representative IFN-γ vs. CellTrace™ violet dilution dot plots for PBMC from control and RB51-vaccinated animals following a 7-day culture with or without RB51 antigen stimulation **(A)** and with or without RB51 antigen and PMA & ionomycin (PI) restimulation **(B)**. Shown are cells gated on CD4^+^ T cells.

Supplementary Figure 3 Proliferative and IFN-γ responses from RB51-specific CD8^+^ T cells following *in vitro* antigen stimulation. Representative IFN-γ vs. CellTrace™ violet dilution dot plots for PBMC from control and RB51-vaccinated animals following a 7-day culture with or without RB51 antigen stimulation **(A)** and with or without RB51 antigen and PMA & ionomycin (PI) restimulation **(B)**. Shown are cells gated on CD8^+^ T cells.

Supplementary Figure 4 Proliferative and IFN-γ responses from RB51-specific γδ T cells following *in vitro* antigen stimulation. Representative IFN-γ vs. CellTrace™ violet dilution dot plots for PBMC from control and RB51-vaccinated animals following a 7-day culture with or without RB51 antigen stimulation **(A)** and with or without RB51 antigen and PMA & ionomycin (PI) restimulation **(B)**. Shown are cells gated on γδ T cells.
