# Supplementary figures and images for: Enhancing the Detection of Brucella-Specific CD4+ T Cell Responses in Cattle via in vitro Antigenic Expansion and Restimulation
Source: Front Immunol. 2020 Sep 2;11:1944. doi: 10.3389/fimmu.2020.01944 (PMC7492661; doi:10.3389/fimmu.2020.01944)

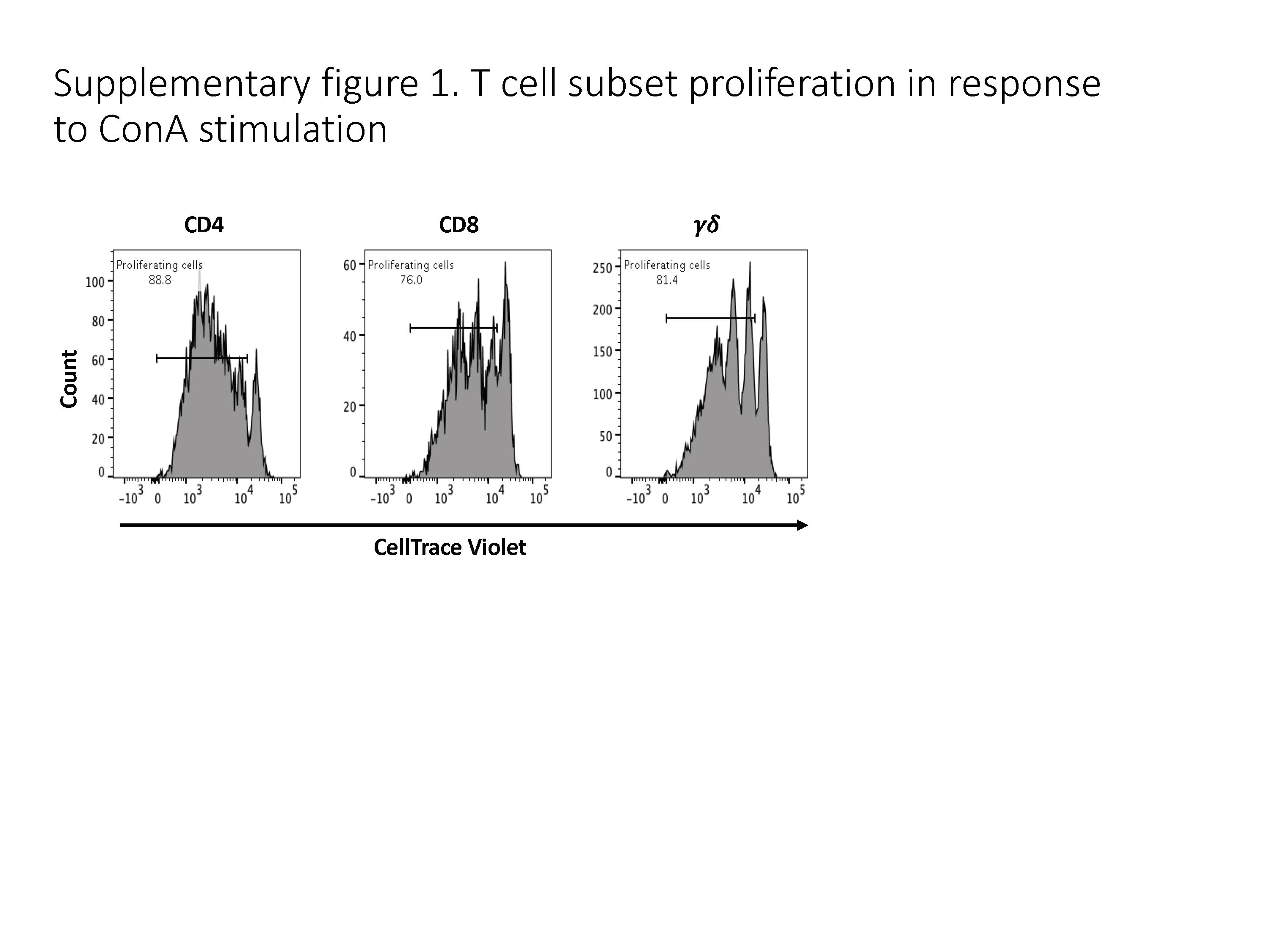

Supplement: Supplementary Figure 1 — T cell subset proliferation in response to ConA stimulation. Representative histograms of CellTrace™ violet dilution for PBMC gated on CD4-, CD8- and γδ-positive cells. [file Image_1.JPEG]

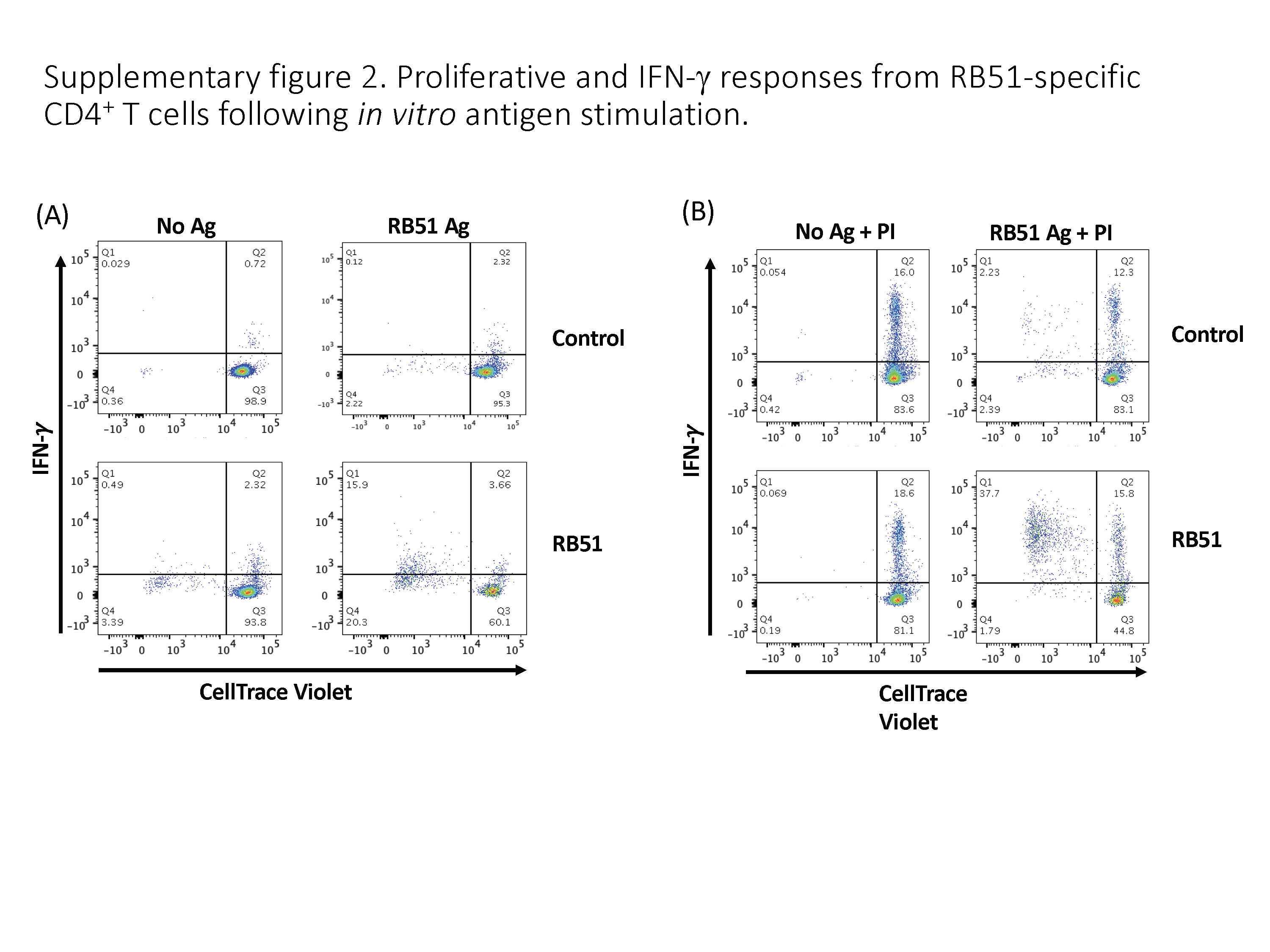

Supplement: Supplementary Figure 2 — Proliferative and IFN-γ responses from RB51-specific CD4+ T cells following in vitro antigen stimulation. Representative IFN-γ vs. CellTrace™ violet dilution dot plots for PBMC from control and RB51-vaccinated animals following a 7-day culture with or without RB51 antigen stimulation (A) and with or without RB51 antigen and PMA & ionomycin (PI) restimulation (B). Shown are cells gated on CD4+ T cells. [file Image_2.JPEG]

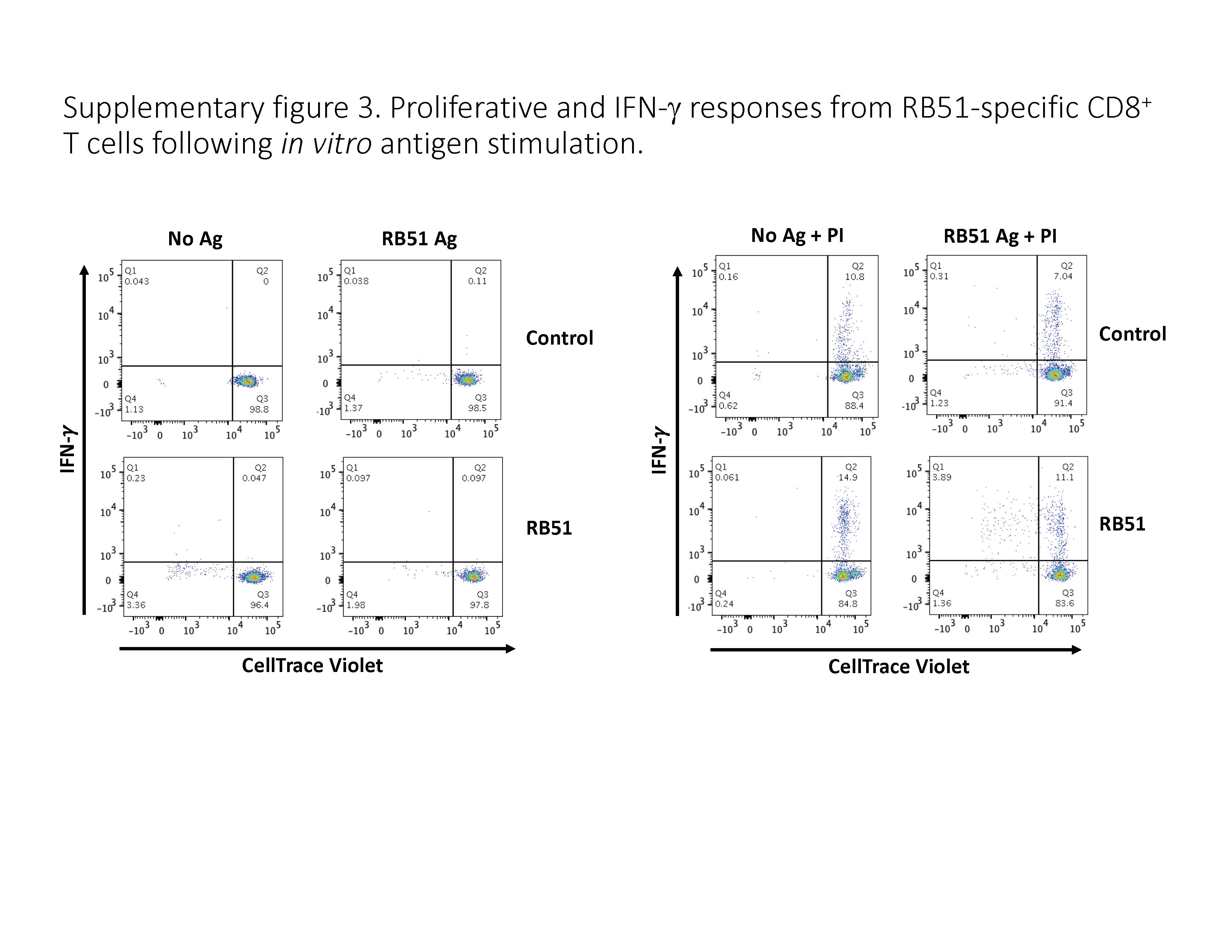

Supplement: Supplementary Figure 3 — Proliferative and IFN-γ responses from RB51-specific CD8+ T cells following in vitro antigen stimulation. Representative IFN-γ vs. CellTrace™ violet dilution dot plots for PBMC from control and RB51-vaccinated animals following a 7-day culture with or without RB51 antigen stimulation (A) and with or without RB51 antigen and PMA & ionomycin (PI) restimulation (B). Shown are cells gated on CD8+ T cells. [file Image_3.JPEG]

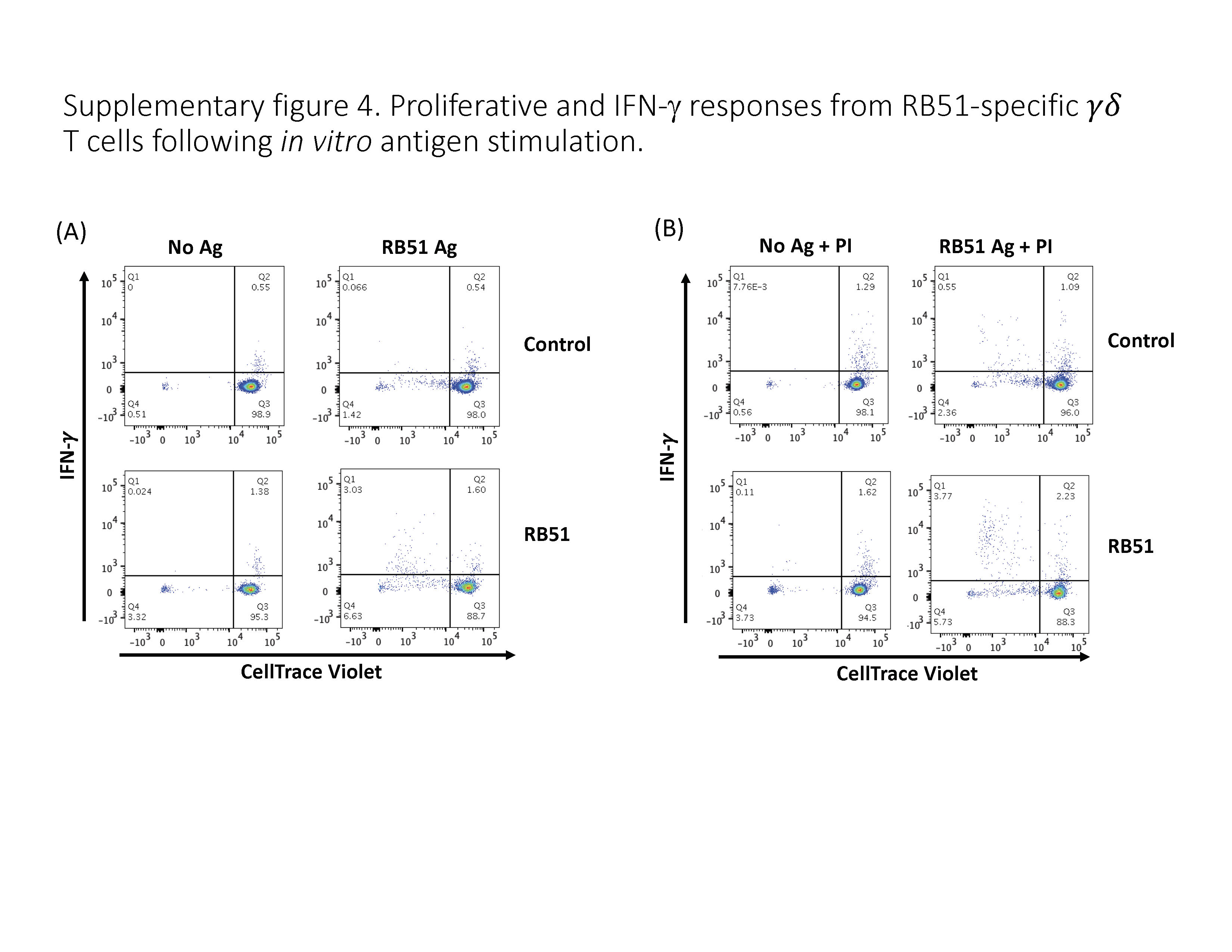

Supplement: Supplementary Figure 4 — Proliferative and IFN-γ responses from RB51-specific γδ T cells following in vitro antigen stimulation. Representative IFN-γ vs. CellTrace™ violet dilution dot plots for PBMC from control and RB51-vaccinated animals following a 7-day culture with or without RB51 antigen stimulation (A) and with or without RB51 antigen and PMA & ionomycin (PI) restimulation (B). Shown are cells gated on γδ T cells. [file Image_4.JPEG]
